# Adolescent Pregnancy: A Case-Series Study of 112 Adolescent Mothers and Their Newborns

**DOI:** 10.7759/cureus.27987

**Published:** 2022-08-14

**Authors:** Estela Kakoo Brioso, Ana I Carvalho, Teresa Caldeira, Ana Vaz, Manuel Cunha

**Affiliations:** 1 Functional Unit of Pediatrics, Children's Department, Hospital De Cascais Dr. José De Almeida, Lisbon, PRT; 2 Area of ​​Medical Pediatrics, Hospital Dona Estefânia, Centro Hospitalar Universitário Lisboa Central, Lisbon, PRT; 3 Functional Unit of Gynecology/Obstetrics, Hospital de Cascais Dr. José de Almeida, Lisboa, PRT; 4 Functional Unit of Neonatology, Children's Department, Hospital de Cascais Dr. José de Almeida, Lisbon, PRT

**Keywords:** social risk, newborns, prematurity, neonatal complications, pregnancy surveillance, adolescent pregnancy

## Abstract

Background

Although declining, adolescent pregnancy remains an important health concern and is associated with adverse maternal and fetal outcomes. We aimed to characterize adolescent pregnancy in a level II hospital and the observed maternal, fetal, and perinatal complications.

Methods

We conducted a case-series study of five-years duration with adolescent mothers and their newborns. We collected sociodemographic, obstetric, and neonatal data through chart review and conducted a comparison analysis between newborns who needed hospitalization and those who did not.

Results

We identified 112 newborns with adolescent mothers. Most pregnancies were unplanned (89.3%) and the start of pregnancy surveillance was late. The most frequent complications were intrauterine growth restriction, oligohydramnios, and threatened preterm labor. Prematurity was found in 9.8% of the newborns and 0.9% had less than 32 weeks at the time of birth. Thirteen newborns (11.6%) needed hospitalization in the neonatal intensive care unit, with three (23%) needing invasive ventilation. The main diagnoses of admission were prematurity, social risk, low birth weight, feeding difficulties, and newborn respiratory distress syndrome. Hospitalization seemed associated with less frequent breastfeeding. Among the hospitalized newborns, there was a high rate of discharge at the care of a relative or an institution (30.8%). Surprisingly, surveillance during pregnancy did not seem to differ between newborns that needed hospitalization and those that did not.

Discussion

Adolescent pregnancy is associated with poor surveillance as well as obstetric and neonatal complications. Newborns of adolescent mothers have a high hospitalization rate, but further investigations are needed to fully understand the contributing factors. The creation of multidisciplinary teams is fundamental for reducing complications, and appropriate reproductive health programs should focus on reducing adolescent pregnancy through better access to education and contraception.

## Introduction

Adolescent pregnancy remains an important public health concern and is responsible for 11% of births worldwide, with more than 90% of these occurring in low-and middle-income countries [1]. In 2020, the adolescent fertility rate in Portugal was 6.7 per 1000 women, and it has been steadily declining over time [2]. The main risk factors for adolescent pregnancy are precocious puberty, obesity, alcohol and drug abuse, mental illness, history of previous physical, emotional, or social abuse, poor emotional support, low social economic status, low level of education, or school dropout [3-4]. Adolescent pregnancy is associated with adverse maternal and fetal outcomes but is difficult to define whether they are associated with maternal age or with the other risk factors listed above [5]. The main fetal complications are intrauterine growth restriction (IUGR) and threatened preterm labor. Regarding perinatal complications, low birth weight (<2500g), preterm delivery, and lower Apgar score are frequently observed [1,5-7].

Therefore, the main goals of this study are to characterize pregnant adolescents in a level II hospital and describe the maternal, fetal, and perinatal complications observed.

## Materials and methods

Design

We conducted a retrospective case series study of adolescent mothers and their newborns born at our hospital.

Inclusion and exclusion criteria

Participants who met the following inclusion criteria were included in the study: mothers whose deliveries occurred between January 2015 and September 2020 and their newborns; and those whose delivery occurred at our hospital delivery room, regardless of the location of prenatal follow-up.

Exclusion criteria were being 18 years or older at the time of delivery or having been delivered at home.

Data collection

Through chart review, we collected data on the sociodemographic characteristics of the parents, pregnancy follow-up, pregnancy-related complications, type of delivery, gestational age at birth, weight at birth, birth-related complications, and need for hospitalization.

Statistical analysis

Statistical analysis was conducted using the IBM® SPSS® Statistics version 22 software (IBM Corp., Armonk, NY). We conducted a descriptive statistical analysis of the sociodemographic variables, as well as pregnancy surveillance variables and neonatal and birth-related characteristics. Participants were also divided into two groups: newborns with a need for hospitalization and newborns with no need for hospitalization in the Neonatal Intensive Care Unit (NICU).

Since the assumptions for parametric tests could not be met in this sample, we used the non-parametric Mann-Whitney U test for comparing continuous variables and the Fisher test for categorical variables. Statistical significance was set at p-values ≤ 0.05.

## Results

Throughout the study period, there were a total of 14,496 deliveries performed at our hospital. Of these, 112 (0.8%) newborns had adolescent mothers at the time of delivery.

Data of parents

The median maternal age was 16.5 years and the median paternal age was 19.0 years (Table 1). The youngest mother was 13 years old and the oldest 17 years old. As for the fathers, the youngest was 14 years old and the oldest was 37 years old. Most fathers (73.3%) were 18 or older. Regarding the mother, a small number (8.8%) were married at the time of birth.

**Table 1:** Sociodemographic characteristics of the parents SD: standard deviation

	Mean	SD	Median	Range
Maternal Age (years)	16.3	0.91	16.5	(13.0 - 17.0)
Paternal Age (years)	19.4	3.43	19.0	(14.0 - 37.0)
	N	%		
Maternal Marital Status				
Single	102	91.1		
Married	10	8.8		
Maternal Education				
4 Years Completed	12	10.7		
6 Years Completed	37	33.0		
9 Years Completed	45	40.2		
12 Years Completed	7	6.3		
Missing Data	11	9.8		
Maternal Country of Birth				
Portugal	81	72.3		
Brazil	12	10.7		
Cape Verde	7	6.2		
Guinea-Bissau	2	1.8		
Bulgaria	2	1.8		
Colombia	1	0.9		
Romania	1	0.9		
Moldavia	1	0.9		
Missing Data	5	4.5		

Data of pregnancy

Most pregnancies were unplanned (89.3%) and the start of pregnancy surveillance was late, at a median gestational age of 13 weeks (Table 2). Less than half of the mothers (48.2%) started surveillance in the first trimester. Seven pregnancies (6.3%) had no surveillance at all.

**Table 2:** Surveillance and complications during pregnancy of the total sample and a comparison of newborns with a need for NICU hospitalization and newborns with no need for NICU hospitalization NICU: neonatal intensive care unit; SD: standard deviation; TORCH: Toxoplasmosis, Other Agents, Rubella, Cytomegalovirus, and Herpes Simplex

		Total		Newborns with a need for NICU hospitalization	Newborns with no need for NICU hospitalization	p-value
		N=112		N=13	N=99	
Start of Pregnancy Surveillance (Weeks of Pregnancy)	Mean ± SD	15.9 ± 7.73		13.2 ± 7.16	16.2 ± 7.77	0.250
	Median (Range)	13 (6 – 37)		11 (6 – 30)	14 (6 – 37)	
		N (%)		N (%)	N (%)	
Start of Pregnancy Surveillance						
1st Trimester		54 (48.2)		7 (53.8)	47 (47.5)	
2nd Trimester		39 (34.8)		2 (15.4)	37 (37.4)	
3rd Trimester		12 (10.7)		1 (7.7)	11 (11.1)	
No Surveillance		7 (6.3)		3 (23.1)	4 (4.0)	
Use of Recommended Vitamin Supplements		85 (75.9)		9 (69.2)	76 (76.8)	0.510
Consumption of Tobacco, Alcohol, or Recreational Drugs During Pregnancy		19 (17.0)		3 (23.1)	16 (16.2)	0.460
Obstetric Complications During Pregnancy						> 0.999
Intrauterine Growth Restriction		11 (9.8)		1 (7.7)	10 (10.1)	
Oligohydramnios		7 (6.3)		2 (15.4)	5 (5.1)	
Threatened Preterm Labor		5 (4.5)		0 (0)	5 (5.1)	
Premature Rupture of Membranes		1 (0.9)		0 (0)	1 (1.0)	
Intrahepatic Cholestasis of Pregnancy		1 (0.9)		0 (0)	1 (1.0)	
Gestational Hypertension		3 (2.7)		1 (7.7)	2 (2.0)	
Fetal Hydronephrosis		1 (0.9)		0 (0)	1 (1.0)	
Other Complications During Pregnancy						0.329
Genital Tract Infections		15 (13.4)		2 (15.4)	13 (13.1)	
Urinary Tract Infection		8 (7.1)		4 (30.8)	4 (4.0)	
TORCH Infection		2 (1.8)		0 (0)	2 (2.0)	
Anemia		7 (6.3)		0 (0)	7 (7.1)	
Thrombocytopenia		1 (0.9)		0 (0)	1 (1.0)	
Hypothyroidism		1 (0.9)		0 (0)	1 (1.0)	
Need for Hospitalization During Pregnancy		20 (17.9)		4 (30.8)	16 (16.2)	0.244

There was a high rate of consumption of alcohol, tobacco, or recreational drugs during pregnancy (N=19; 17.0%).

The most frequent obstetric complications were intrauterine growth restriction (IUGR), oligohydramnios, and threatened preterm labor (9.8%, 6.3%, and 4.5%, respectively). Infections were also common complications: 13.4% of genital tract infections and 7.1% of urinary tract infections. Twenty mothers (17.9%) needed to be hospitalized during the course of pregnancy (Table 2).

Data of the newborns

Normal vaginal delivery was the most frequent type of delivery (77.7%), followed by operative vaginal delivery (11.6%). Twelve newborns (10.7%) needed cesarean delivery (Table 3).

**Table 3:** Neonatal and birth-related characteristics of the total sample and comparison between newborns with a need for NICU hospitalization and newborns with no need for hospitalization NICU: neonatal intensive care unit; SD: standard deviation

		Total	Newborns with a need for NICU hospitalization	Newborns with no need for NICU hospitalization	p-value
		N=112	N=13	N=99	
Birth Weight (g)	Mean ± SD	3145 ± 542.8	2546 ± 739.5	3223 ± 461.4	0.001
	Median (Range)	3175 (950 – 4535)	2885 (950 – 3410)	3215 (2075 – 4535)	
Gestational Age (weeks)	Mean ± SD	38.5 ± 1.87	35.9 ± 3.73	38.8 ± 1.13	0.013
	Median (Range)	39 (29 – 41)	36 (29 – 40)	39 (36 – 41)	
		N (%)	N (%)	N (%)	
Type of Delivery					
Normal Vaginal Delivery		87 (77.7)	11 (84.6)	76 (76.8)	
Operative Vaginal Delivery		13 (11.6)	0 (0)	13 (13.1)	
Cesarean Delivery		12 (10.7)	2 (15.4)	10 (10.1)	
Gender					
Male		47 (42.0)	5 (38.5)	42 (42.4)	
Female		65 (58.0)	8 (61.5)	57 (57.6)	
Size for Gestational Age					
Small for Gestational Age		12 (10.7)	1 (7.7)	11 (11.1)	
Appropriate for Gestational Age		96 (85.7)	11 (84.6)	85 (85.9)	
Large for Gestational Age		4 (3.6)	1 (7.7)	3 (3.0)	
Need for Resuscitation		5 (4.5)	1 (7.7)	4 (4.0)	
Birth-related Complications					
Shoulder Dystocia		3 (2.7)	0 (0)	3 (3.0)	
Birth Fracture of the Clavicle		1 (0.9)	0 (0)	1 (1.0)	
Cephalohematoma		1 (0.9)	0 (0)	1 (1.0)	

At birth, the median gestational age was 39 weeks. Prematurity was found in 9.8% of the newborns and 0.9% had less than 32 weeks at the time of birth. The median birth weight was 3175 g and most newborns were appropriate for gestational age (85.7%). The lowest documented birth weight was 959 g and the highest was 4535 g. Ten (8.9%) newborns had a low birth weight (< 2500 g), of which just one had an extremely low birth weight (<1000 g). Four newborns were macrosomic (> 4000 g) and the other newborns had a normal birth weight (N=98; 87.5%). Most newborns were females (58.0%) (Table 3).

Although there were 22 newborns with infectious risk, none developed early-onset neonatal sepsis. Sixteen had developed hyperbilirubinemia that required phototherapy. Three newborns presented respiratory distress and required invasive mechanical ventilation.

Three newborns had shoulder dystocia, one had a birth fracture of the clavicle, and one had a cephalohematoma. There was a need for resuscitation in five newborns and 11.6% (N=13) of the newborns needed hospitalization in NICU. Of these, three (23%) needed intensive care.

Taking into account the newborns who required hospitalization in NICU, the lowest gestational age was 29 weeks and the median gestational age was 36 weeks (Table 3). The mean length of stay in the NICU was 18 days, but one newborn was hospitalized for 98 days. The main diagnoses of admission were prematurity (N=5), social risk (N=4), low birth weight (N=3), feeding difficulties (N=2), newborn respiratory distress syndrome (N=2), coarctation of the aorta (N=1), and hydrocephalus after intraventricular hemorrhage (N=1).

Data of hospitalized newborns

As expected, newborns that needed hospitalization had lower birth weight (p-value = 0.001) and gestational age (p-value = 0.013) than those with no need for hospitalization in the NICU (Table 3).

Surveillance during pregnancy did not seem to differ between newborns that needed hospitalization and those that did not: gestational age at the first appointment and the number of appointments were similar (p-value = 0.250 and p-value = 0.782, respectively) (Table 2). Similarly, we did not find any differences in the use of recommended vitamin supplements (p-value = 0.510), consumption of tobacco, alcohol, or recreational drugs (p-value = 0.460), complications throughout the pregnancy (p-value > 0.999 and p-value = 0.329), or cesarean rate (p-value = 0.629).

Of the newborns that were hospitalized, only 69.2% were discharged to their parents. The others were discharged under the care of a relative (15.4%) or an institution (15.4%).

Hospitalization seemed associated with less frequent breastfeeding (p-value = 0.008). Only 46.2% of the newborns that required hospitalization were breastfed at the time of discharge; in comparison, most newborns that did not need hospitalization in NICU were breastfed at the time of discharge (81.8%). Although an interesting result, its interpretation is made difficult due to the low number of hospitalized newborns and the retrospective nature of this study.

## Discussion

Over the last decades, many studies have been published about adolescent pregnancy, although most of these were developed in low-income countries, where the problem seems to be more prominent [6]. In our study, 0.8% of all pregnancies were in adolescence, slightly below the national average (1%) [8]. The youngest mother was 13 years old and as expected, most of these pregnancies were unplanned (89.3%).

Knowledge gaps and misconceptions on where to obtain contraception and how to use it might partially contribute to the high rate of adolescent pregnancy. Additionally, adolescents may lack the autonomy to ensure the correct and consistent use of a contraceptive method [9]. To address this problem, the World Health Organization recommends improving access to information and contraception and retaining adolescents in school until the secondary level, among other measures, to reduce pregnancy before the age of 20 years [10]. In Portugal, adolescents have access to free reproductive health appointments in primary care centers, as well as free contraception, such as condoms and birth control pills [11]. Despite this, adolescent pregnancy rates remain high, which might indicate that other factors not addressed may also play a role.

In our study, the start of pregnancy surveillance was predominately late and the rate of substance consumption during the pregnancy was 17%. These confounders do not allow us to state that the adverse obstetric, fetal, and perinatal outcomes described are due to biological factors rather than to discrepancies between nutrition, social context, emotional support, and prenatal care between adolescent and adult mothers [3-4]. The late start of pregnancy surveillance may be a surrogate for a low level of health education. In fact, most mothers (83.9%) had nine years or less of education, which is low considering their age.

The most important obstetric complications found in our study were urogenital tract infections (20.5%), IUGR (9.8%), oligohydramnios (6.3%), and threatened preterm labor (4.5%). Similar rates were found in other studies [1,3-5]. Cesarean section was performed in 10.7% of our cases, a rate lower than the cesarean section rate at our hospital (27%) and the national cesarean section rate (34.2%) [2]. These data confirm the expected lower rate of cesarean section in pregnant adolescents [3,5].

Between 2015 and 2020, the global percentage of newborns with low birth weight in Portugal was 7.9 to 9.0% but was 8.2% to 10.4% among pregnant adolescents [12]. In our study, low birth weight occurred in 10.7% of all pregnancies, a rate slightly higher than the national data [12]. In the same period, premature births occurred in 6.8% to 8.1%, comparable to the percentage found in adolescent pregnancy (6.6% to 8.6%). We found prematurity in 9.8% of the newborns, a higher percentage than national data [12].

In our study, 11.6% of newborns of adolescent mothers needed hospitalization in the NICU. As expected, they had lower birth weight and lower gestational age than non-hospitalized newborns, but no differences were found in pregnancy surveillance, consumption of substances, or complications throughout the pregnancy.

Among the hospitalized newborns, more than 30% were discharged under the care of an institution or a relative other than the parents. We speculate that low parenting skills, particularly in families with less social support, might explain the high rate of discharge to the care of people other than the parents.

Although having found high rates of obstetric and neonatal complications, as well as poor pregnancy surveillance, in our sample, the lack of a comparison group of adult mothers does not allow us to definitely state that these rates are associated with adolescence. Likewise, the retrospective nature of our study design does not permit us to infer causality relations between our data. Our study raises questions to be answered with follow-up investigations, particularly prospective studies comparing adolescent and adult mothers to fully understand which risk factors found in adolescents contribute to adverse outcomes and plan interventional studies to ascertain the benefit of addressing them.

## Conclusions

Adolescent pregnancy is associated with important clinical and social risks. Newborns of adolescent mothers have a high hospitalization rate. The low pregnancy surveillance rates might play a role but further studies are needed to explain these findings.

Although declining, adolescent pregnancy is still a public health problem that needs to be addressed, and the creation of multidisciplinary teams, including healthcare professionals and social workers, is fundamental for reducing both maternal and perinatal complications. Appropriate reproductive health education programs in schools should be available. Adolescents need access to youth-friendly reproductive health services and support from parents and other trusted adults, who can play an important role in helping them make healthy choices about relationships, sex, and birth control.
